# Laser-Based 3D Body Scanning Reveals a Higher Prevalence of Abdominal Obesity than Tape Measurements: Results from a Population-Based Sample

**DOI:** 10.3390/diagnostics13152594

**Published:** 2023-08-04

**Authors:** Robert P. Kosilek, Till Ittermann, Dörte Radke, Sabine Schipf, Matthias Nauck, Nele Friedrich, Henry Völzke

**Affiliations:** 1 Institute for Community Medicine, University Medicine Greifswald, 17475 Greifswald, Germany; 2 Institute of General Practice and Family Medicine, University Hospital, LMU Munich, 80336 Munich, Germany; 3 Institute for Medical Information Processing, Biometry and Epidemiology (IBE), LMU Munich, 81377 Munich, Germany; 4 German Center for Diabetes Research, Partner Site Greifswald, 17475 Greifswald, Germany; 5 Institute for Clinical Chemistry and Laboratory Medicine, University Medicine Greifswald, 17475 Greifswald, Germany; 6 German Center for Cardiovascular Research, Partner Site Greifswald, 17475 Greifswald, Germany

**Keywords:** metabolic syndrome, obesity, anthropometry, body weights and measures, diagnostic techniques and procedures

## Abstract

Background: The global obesity epidemic is a major public health concern, and accurate diagnosis is essential for identifying at-risk individuals. Three-dimensional (3D) body scanning technology offers several advantages over the standard practice of tape measurements for diagnosing obesity. This study was conducted to validate body scan data from a German population-based cohort and explore clinical implications of this technology in the context of metabolic syndrome. Methods: We performed a cross-sectional analysis of 354 participants from the Study of Health in Pomerania that completed a 3D body scanning examination. The agreement of anthropometric data obtained from 3D body scanning with manual tape measurements was analyzed using correlation analysis and Bland–Altman plots. Classification agreement regarding abdominal obesity based on IDF guidelines was assessed using Cohen’s kappa. The association of body scan measures with metabolic syndrome components was explored using correlation analysis. Results: Three-dimensional body scanning showed excellent validity with slightly larger values that presumably reflect the true circumferences more accurately. Metabolic syndrome was highly prevalent in the sample (31%) and showed strong associations with central obesity. Using body scan vs. tape measurements of waist circumference for classification resulted in a 16% relative increase in the prevalence of abdominal obesity (61.3% vs. 52.8%). Conclusions: These results suggest that the prevalence of obesity may be underestimated using the standard method of tape measurements, highlighting the need for more accurate approaches.

## 1. Introduction

At an estimated worldwide prevalence of about 25%, metabolic syndrome is an increasing global public health concern with significant implications for both individual health and healthcare systems [1]. The syndrome comprises abdominal obesity, dyslipidemia, impaired glucose metabolism and hypertension [2]. Treatment typically consists of lifestyle changes and symptomatic pharmacotherapy when no underlying cause can be identified [3]. Abdominal adipose tissue seems to be of pathophysiological importance for the development of obesity-related comorbidities. Diagnostic criteria of metabolic syndrome are therefore based on the classification of abdominal obesity by waist circumference, among others.

Three-dimensional (3D) body scanning is a relatively new method for body measurements [4,5]. A 3D digital body model is commonly created using structured light involving specialized cameras, sensors, and software [6,7]. This technology offers several advantages over manual tape measurements: it is fast, accurate, comprehensive, and non-invasive. Applications range from fashion design to ergonomics and healthcare. The validity and reliability of body scanning technology compared to manual measurements was repeatedly demonstrated [8,9,10,11,12,13,14,15].

This study was conducted to validate the first wave of 3D body scan data from a German population-based cohort for future research projects and explore clinical implications of this technology in the context of metabolic syndrome.

## 2. Methods

### 2.1. Study Design and Population

This cross-sectional study utilized data from the SHIP-TREND cohort of the Study of Health in Pomerania (SHIP), a representative population-based, longitudinal observational study of adult residents in northeastern Germany. The SHIP study design, protocol, and sampling methods have been described in previous publications [16,17,18]. It was reviewed and authorized by the ethics committee of the University of Greifswald and adheres to the Declaration of Helsinki. All subjects gave written informed consent prior to participation. Participants included in this sample were examined between February 2011 and August 2012 in Greifswald, Germany.

For the SHIP-TREND cohort, 4420 out of 8826 eligible individuals participated in the baseline exam (TREND-0) between 2008 and 2012. Stationary laser-based 3D body scanning was introduced towards the end of baseline data collection for the SHIP-TREND cohort as an optional examination, thus limiting the sample size. For inclusion in this analysis, participants must have completed manual anthropometry and 3D body scanning (*n* = 355), standardized interviews, and provided blood samples. Pregnancy, consuming disease indicated by BMI < 18 kg/m^2^, severe physical deformities, or missing interview variables (*n* = 1) at the time of examination were used as exclusion criteria. The final analytical sample consisted of 354 SHIP-TREND-0 study participants (160 male, 194 female).

### 2.2. Data

All measurements and use of technical equipment were completed in a standardized method by trained personnel, as previously described [16,17,19]. Details on all variables, including survey questions in the German language, can be found online in the SHIP data dictionary [20]. Sociodemographic characteristics, medical history, current medication, and lifestyle indicators were acquired through standardized computer-assisted interviews.

The following laboratory parameters were included in the analyses: plasma glucose, total/LDL/HDL cholesterol and triglycerides as part of the definition of the metabolic syndrome, and hemoglobin A1c (HbA1c) as an indicator of long-term glycemic control for descriptive statistics. Blood samples were drawn from the cubital vein in the supine position, and serum aliquots were prepared for immediate analysis and for storage at −80 °C. Levels of LDL and HDL cholesterol were determined using the liquid selective detergent method (Dimension Vista 500 analytical system, Siemens AG, Erlangen, Germany). Glucose, total cholesterol, and triglyceride levels were assessed using photometric methods (Dimension Vista; Flex reagent cartridge; Dade Behring Ltd., Milton Keynes, UK). HbA1c was measured using high-performance liquid chromatography with spectrophotometric detection (Diamat Analyzer; Bio-Rad, Munich, Germany).

Bioelectrical impedance analysis was performed using the Nutriguard M System (Data Input GmbH, Darmstadt, Germany). Body fat percentage, automatically calculated from lean mass, body weight, and corrected for body water, was used for analyses.

Four manual tape measurements (body height, circumferences of waist, hip, and right upper arm) were available for comparison with 3D body scan measurements. Body size was determined using a SOEHNLE body length measuring device to an accuracy of 1.0 cm in an upright position without shoes. Weight was measured with a SOEHNLE-S20 personal scale to an accuracy of 100 g while wearing underwear and leg clothes. Hip and waist circumference were measured after the additional removal of constricting garments (e.g., medical corsets) using an inelastic tape measure to an accuracy of 0.5 cm, with a full body used to check the position of the tape. Waist circumference was measured at the narrowest point between the last rib and the highest point of the iliac crest. Hip circumference was measured at the point of the largest circumference between the highest point of the iliac crest and the crotch. Right upper arm circumference was measured prior to blood pressure measurements for determination of the cuff size. All parameters were obtained according to standardized written instructions in line with WHO recommendations [17,19].

**Figure 1:**
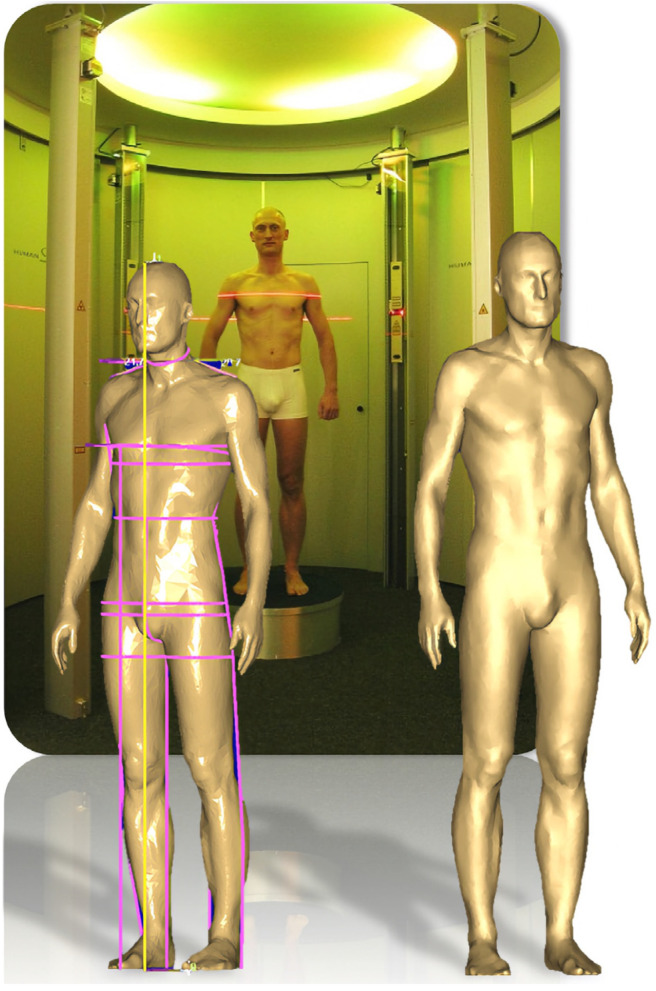
Composite sample image of the laser-based 3D body scanner Vitus Smart XXL and a resulting 3D model with an overlay of various anthropometric measures (e.g., waist and hip circumference). Copyright Human Solutions GmbH, Kaiserlautern, Germany. Printed with permission.

Three-dimensional body scan measurements were automatically obtained using a laser-based three-dimensional body surface scanner (3D Bodyscanner VITUS Smart XXL) with Anthroscan software for data processing (both Human Solutions GmbH, Kaiserslautern, Germany). This device is compliant with the DIN EN ISO 20685 norm (“3-D scanning methodologies for internationally compatible anthropometric databases”), so landmarks are based on existing anthropometric standards. Figure 1 shows a composite sample image of the body scanner and a resulting 3D model with measurements. Subjects assumed a standardized position for the scanning procedure, standing upright in a natural posture with feet shoulder-width apart and arms slightly away from the body, wearing only underwear and a bathing cap to compress the hair. Landmarks for measurement are specified by the manufacturer and autodetected by the proprietary Anthroscan software. The variables were visualized and subsequently assessed regarding any known or theoretical connection to the metabolic syndrome. For both waist and hip circumference, four body scan variables in close proximity to each other were available for comparison with corresponding tape measurements, out of which the best match was selected. Variables without direct medical relevance to this study, as well as 14 manually programmed variables, were excluded from analyses. Out of 167 initially available body scan variables including weight, 28 were retained after the selection process. Three sagittal diameter variables were obtained by calculating the respective differences from a central frontal and dorsal landmark to the rear boundary of the body scanning area (neck diameter at base, chest diameter, diameter at maximum belly circumference). Eight implausible individual body scan values (extreme outliers, <0.001% of measurements) were identified using scatterplot graphs and set to missing for available case analysis. For thigh circumference, upper arm circumference, and upper arm diameter, agreement between the left and right-side measurements was assessed by calculating Pearson’s r. For further analyses, only the right-side variables were included.

Body mass index (BMI, weight in kilograms divided by squared height in meters) and waist to height ratio (WHtR, waist circumference in cm divided by height in cm) were used as composite measures and calculated using both tape and body scan measurements for comparison.

### 2.3. Metabolic Syndrome Classification

The metabolic syndrome was defined according to the harmonized IDF diagnostic criteria [2], requiring ≥3 of the following 5 criteria: (I) waist circumference greater than 94 cm for males and 80 cm for females; (II) plasma glucose greater than 5.6 mmol/L at more than 8 h of fasting; (III) HDL cholesterol lower than 1.0 mmol/L for males and 1.3 mmol/L for females; (VI) triglycerides greater than 1.7 mmol/L; (V) hypertension as evidenced by blood pressure greater than 130/85 mm Hg; targeted pharmacotherapy is an alternate indicator for criteria II–V. Due to the fact that 28% of subjects did not provide fasting blood samples, we additionally defined glucose of greater than 8 mmol/L at less than 8 h of fasting as pathological, which resulted in one additional case of metabolic syndrome.

### 2.4. Statistical Analysis

Statistical analyses were completed using Stata IC 14 (Stata Corp., College Station, TX, USA). *p*-values smaller than 0.05 were considered statistically significant. The study sample was stratified by gender for analyses. Categorical data are presented using absolute and relative frequency, and continuous data are presented using median and interquartile range. Missing data affected only a few individual observations (max. 5 missing values in 13 variables) and was handled by available case analysis. The Mann–Whitney U test and Fisher’s exact test were used to evaluate differences in continuous and categorical data stratified by gender and metabolic syndrome with Bonferroni correction for five comparisons groups. The agreement of manual and automatic measurements was assessed using Pearson’s correlation coefficient and by comparing differences using the sign test. Additionally, we produced Bland–Altman plots for the variables with the best agreement using the batplot package for Stata [21,22,23]. The agreement between manual and automatic measurements regarding the classification of the metabolic syndrome, abdominal obesity, BMI, and WHtR was assessed by calculating Cohen’s kappa. An exploratory correlation analysis of body scan data and metabolic syndrome components, as well as body fat percentage was conducted using Spearman’s rho.

## 3. Results

### 3.1. Baseline Characteristics

Baseline characteristics of the study sample are shown in Table 1. The age distribution did not differ significantly between men and women. The metabolic syndrome had an overall prevalence of 31% according to IDF criteria, with a significant gender-specific imbalance (males 44.4% vs. females 20.1%). Overweight and obesity were frequent in this sample, with 63% of all subjects showing a BMI ≥ 25 kg/m^2^. There were no significant differences regarding sociodemographic and lifestyle characteristics, such as education, relationship status, physical activity, or dietary habits. Extended baseline characteristics with laboratory parameters and comparisons by metabolic syndrome classification are shown in the Supplementary Material (Table S1).

### 3.2. Agreement of Manual and Automatic Measurements

All calculations regarding the agreement of tape and body scan measurements are presented in the Supplementary Material (Table S2), with the main results shown in Table 2. Based on these calculations, as well as a visual comparison of the 3D body scan landmarks with tape measurement positions, the body scan circumference variables at the high waist and middle hip positions show the best agreement with the corresponding tape measurements. Body scan measurements show small, positive deviations from manual measurements with very high correlation coefficients ranging from 0.80 to 0.99. Bland–Altman plots for the relevant variables revealed a range of mean differences between 0.19 and 3.16 cm and between 3 and 7% outliers beyond the 95% limits of agreement, which are provided in the Supplementary Material (Table S3).

### 3.3. Practical Implications

**Figure 2:**
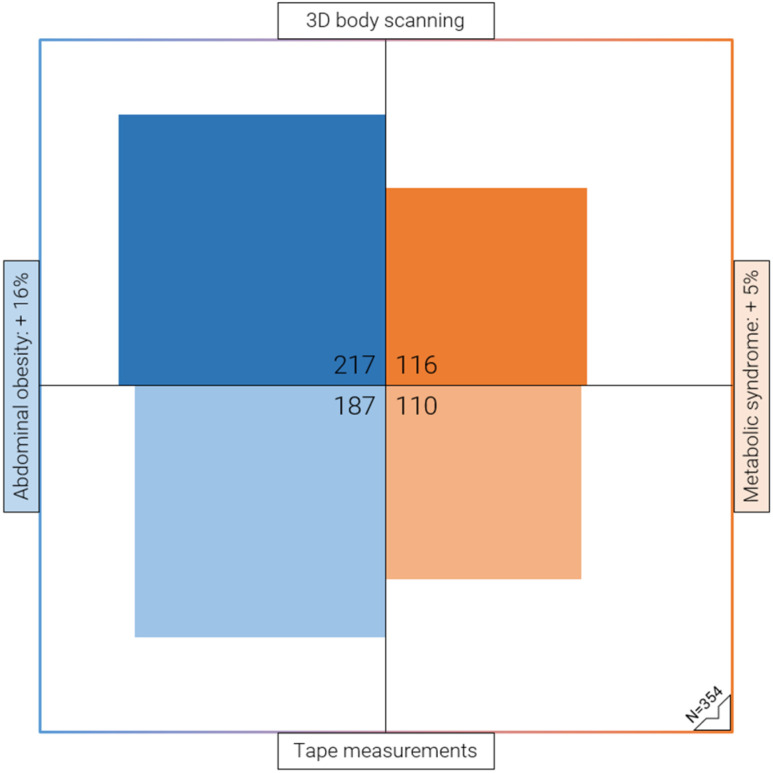
Proportional area chart illustrating the high prevalence of abdominal obesity (61%) in the study sample, as determined by 3D body scanning. Absolute and relative increase of classified cases of abdominal obesity (blue) and metabolic syndrome (orange) by 3D body scan vs. tape measurements of waist circumference, according to the European IDF criteria. Each quadrant represents the total sample size of *n* = 354.

We compared the classification agreement of tape and body scan measurements regarding abdominal obesity and the metabolic syndrome and found very high agreement with Cohen’s κ values ranging from 0.79 to 0.96. However, 3D body scanning resulted in 30 additional cases of abdominal obesity (217 vs. 187 of 354) (κ = 0.79, *p* < 0.05) and 6 additional cases of metabolic syndrome (116 vs. 110 of 354) (κ = 0.96, *p* < 0.05) over tape measurements (Figure 2). Classification of central obesity by WHtR (cutoff ≥ 0.5) resulted in 9 additional cases (217 vs. 208 of 353) (κ = 0.88, *p* < 0.05). For BMI classification, body scanning resulted in diverging results in 17 out of 352 cases (κ = 0.93, *p* < 0.05).

### 3.4. Anthropometric Measurements

A comprehensive comparison of central and peripheral body scan measurements by gender and classification of metabolic syndrome is shown in the Supplementary Material (Table S4). Most of these measurements differ significantly in all comparisons. Body height differs significantly in women with metabolic syndrome but not in men.

### 3.5. Metabolic Syndrome

Correlation analyses of body scan measurements with metabolic syndrome components are presented in the Supplementary Material (Table S5). Most measurements show moderate correlation with the metabolic syndrome, the sum of metabolic syndrome components without waist circumference, and hypertension. For elevated glucose, low HDL cholesterol, and elevated triglycerides, there was either no significant or only weak correlation with body scan measurements. Almost all measurements show very high correlation with body fat percentage in women and a slightly lower correlation in men.

## 4. Discussion

In this study, we assessed the validity of 3D body scanning technology compared to manual tape measurements in a population-based sample and explored clinical implications.

### 4.1. Body Scan and Manual Tape Measurements Show Excellent Agreement

We were able to confirm excellent agreement of tape and body scan measurements obtained in this study cohort, enabling further analyses with data from follow-up examinations. Consistent with other studies, we found that body scan parameters are slightly larger than the corresponding tape measurements [8,9,13,14]. This might be due to the constriction of soft tissue via tape tension or subject behavior, such as holding their breath or pulling in the stomach, during measurements, as previously described [13]. Body scan measurements might, accordingly, give a better estimate of the true circumference at the examined location.

### 4.2. Body Scanning Reveals a Higher Prevalence of Abdominal Obesity

Using 3D body scanning to classify abdominal obesity according to the European IDF cutoff resulted in 30 additional cases, representing a prevalence increase from 52.8% to 61.3% (217 vs. 187 of 354) in our sample. Since abdominal obesity is one of five diagnostic criteria for metabolic syndrome, its prevalence also increased from 31.1% to 32.8% (116 vs. 110 of 354) for a subset of participants that would not otherwise fulfil the diagnostic criteria based on tape measurements. This finding has important epidemiological and clinical implications. For epidemiological considerations regarding the prevalence of obesity, it raises the question of whether the standardized measuring methods, the estimates of prevalence, or the according cut-off values for abdominal obesity need to be re-evaluated. For clinical considerations, accurate diagnosis of obesity is essential for identifying individuals who may benefit from health interventions.

### 4.3. Other Literature

Jaeschke et al. published a comparable study regarding the accuracy of body scan measurements obtained using the same device, with mostly comparable results, but a much smaller sample size of 60 subjects [13]. Petrescu et al. analyzed the association of a broad selection of body scan measurements—also acquired using the same device—with type 2 diabetes mellitus in a comparable sample size (*n* = 357). Only weak correlations were found, which is consistent with our results [24]. There are a few other studies that analyzed the association of the metabolic syndrome and metabolic risk factors with composite measurements calculated from multiple body scan variables, which are not directly comparable to our study due to diverging cut-offs for metabolic syndrome criteria and the use of cross-sectional area variables from body scanning [25,26,27].

### 4.4. Risk Assessment

We could not identify any specific body measurements that showed significantly better correlation with metabolic syndrome components than others, including those that are more easily obtained by body scanning, such as sagittal diameters. Thigh and arm measurements exhibit only poor correlation with metabolic syndrome components, confirming the importance of central obesity for metabolic syndrome. Other studies have also used body scanning to evaluate the association of body shape and metabolic syndrome in international comparisons and came to a similar conclusion, emphasizing this technology’s advantage of taking individual variability of body shape into account. [28,29] Body fat percentage is also associated with metabolic syndrome and showed excellent correlation with anthropometric measurements, indicating that body scanning could be used to estimate body fat percentage [30].

### 4.5. Strengths and Limitations

This study provides a comprehensive assessment of 3D body scan measurements and their association with metabolic risk factors and thus makes a valuable contribution to the field of obesity research and imaging technology. One potential limitation might arise from the relatively small sample size and the fact that body scanning was an optional examination, which may have introduced selection bias. However, based on the distribution of characteristics in the study population compared to similar publications using SHIP-TREND-0 data, there does not appear to be a relevant selection effect beyond a slightly larger proportion of females [31]. Second, both systematic and random errors can occur with the use of 3D body scanning technology. To address this, we have used carefully selected, pre-programmed, autodetected measurements for this analysis. The automatic setup minimizes random or systematic errors that might arise from manual placement of landmarks on the 3D models, as well as intra- or interobserver bias. While autodetection can occasionally result in erroneous measurements, it only occurred in less than 0.001% of body scan measurements (8 out of 9912 values from 28 variables in 354 observations), and we eliminated extreme outliers from the dataset. In contrast, the accuracy and reliability of manual tape measurements has been shown to be limited mostly by systematic error due to interobserver differences and the dimensions of the measurements, with larger measurements appearing to be more reliable [32,33,34,35,36,37]. Therefore, it is reasonable to assume that the advantages of body scanning technology outweigh potential problems arising from occasional outliers. Lastly, since this is a cross-sectional study design aimed to explore associations, no longitudinal modeling of outcomes, such as cardiovascular events, was performed. Future studies on this topic might pursue a longitudinal design to identify novel predictive anthropometric markers from 3D body scanning.

### 4.6. Perspectives

The metabolic syndrome leads to a reduced quality of life and an increased risk for metabolic and cardiovascular disorders if left untreated. An easy and reliable screening method is needed to identify individuals at high risk for disease progression and complications at an early stage. The utility of body measurements for risk assessment is well known [31]. However, the procedure of obtaining body measurements beyond height and weight is rarely standardized if it is done at all. Body scanning offers unobtrusive, fast, and reliable measurements, and physicians might be more inclined to use these results for risk assessment if the data were easily available. A recent study presented a promising approach, in which 3D body scanning was used to classify body shapes into several categories for risk assessment [38]. It is also important to consider potential risks associated with capturing detailed information about a person’s shape and size using this technology, such as data ownership, security and privacy concerns, adverse psychological effects with regard to body image, or inaccurate assessments due to technical limitations. However, with adequate mitigation strategies for these issues, 3D body scanning could be used as a versatile clinical tool, e. g., for the identification of postural problems in physical medicine, visualization and documentation of treatment results in orthopedics or reconstructive surgery, or progress documentation in obesity treatment [6].

## 5. Conclusions

This study provides comprehensive anthropometric measurements obtained from 3D body scanning in a population-based sample. Our findings suggest that the prevalence of obesity may be underestimated using the standard method of tape measurements, highlighting the need for more accurate approaches in order to improve diagnosis, identify at-risk individuals, and facilitate early interventions to prevent obesity-related complications.

## Figures and Tables

**Table 1:** Baseline characteristics of the study population.

	Males (*n* = 160; 45.2%)			Females (*n* = 194; 54.8%)		
Variable	Median/IQR or %/*n*					
Age (years)	44.5	/	22.0	45.0	/	19.0
BMI (kg/m^2^)	27.5	/	5.11	25.1	/	6.94
BMI < 25 kg/m^2^	21.9%	/	35	49.5%	/	96
BMI 25–29.9 kg/m^2^	50.6%	/	81	29.9%	/	58
BMI 30–34.9 kg/m^2^	21.9%	/	35	16.0%	/	31
BMI ≥ 35 kg/m^2^	5.6%	/	9	4.6%	/	9
Waist circumference (cm)	95.5	/	14.7	80.1	/	16.9
Abdominal obesity ^1^	56.3%	/	90	50.0%	/	97
WHtR	0.54	/	0.09	0.49	/	0.11
Body fat percentage	23.4	/	6.60	32.7	/	10.7
Metabolic syndrome ^1^	44.4%	/	71	20.1%	/	39

BMI: body mass index; WHtR: waist to height ratio. (^1^) IDF European diagnostic criteria (Alberti et al., Circulation 2009 [2]).

**Table 2:** Comparison of tape and 3D body scan measurements.

		Tape Measurements	3D Body Scan Measurements	Delta ^1^	Correlation ^2^
Height	M	179.0/8.0	179.3/9.0	0.3/1.8	0.990
	F	165.0/9.0	165.2/8.3	0.6/1.1	0.990
Right upper arm circ.	M	31.0/4.0	31.7/3.2	0.9/2.2	0.801
	F	28.0/4.6	28.7/3.8	1.0/2.0	0.896
Waist circumference	M	95.5/14.7	98.4/15.6	2.8/3.4	0.968
	F	80.1/16.9	82.6/17.6	3.1/3.4	0.975
Hip circumference	M	99.1/9.3	100.0/13.3	2.1/6.3	0.888
	F	98.5/13.9	98.2/15.5	1.0/4.2	0.946

M: male subjects (*n* = 160); F: female subjects (*n* = 194). All variables measured in centimeters, results presented as median and interquartile range. (^1^) Difference body scan measurement—tape measurement, sign test *p* < 0.05 for all results. (^2^) Pearson’s *r*, *p* < 0.05 for all results.

## Data Availability

SHIP data are publicly available for scientific and quality control purposes on request based on a standardized data application procedure (https://transfer.ship-med.uni-greifswald.de/FAIRequest (accessed on 31 July 2023)). The informed consent obtained from the participants of the study does not cover data storage in public databases.

## Supplementary Materials

The following supporting information can be downloaded at: https://www.mdpi.com/article/10.3390/diagnostics13152594/s1, Table S1: Extended baseline characteristics with group comparisons. Table S2: Comparison of tape and 3D body scan measurements. Table S3: Bland-Altman plots. Table S4: 3D body scan measurements by gender and metabolic syndrome classification. Table S5: Correlation of 3D body scan measurements with metabolic syndrome components.
