# Supplementary material for: Laser-Based 3D Body Scanning Reveals a Higher Prevalence of Abdominal Obesity than Tape Measurements: Results from a Population-Based Sample
Source: Diagnostics (Basel). 2023 Aug 4;13(15):2594. doi: 10.3390/diagnostics13152594 (PMC10417794; doi:10.3390/diagnostics13152594)
Supplement: Supplementary file 1 [file diagnostics-13-02594-s001.zip › diagnostics-2464808-supplementary.pdf]

Laser-based 3D body scanning reveals a higher prevalence of abdominal obesity than tape measurements: Results from a population-based sample

Kosilek RP et al., Diagnostics 2023

Supplementary material:

Table S1: Extended baseline characteristics with group comparisons

Table S2: Comparison of tape and 3D body scan measurements

Table S3: Bland-Altman plots

Table S4: 3D Body scan measurements by gender and metabolic syndrome classification

Table S5: Correlation of 3D body scan measurements with metabolic syndrome components

Table S1: Extended baseline characteristics with group comparisons

Gender	Male: N=160 (45.2%)		Female: N=194 (54.8%)		Comparisons†			
Metabolic syndrome	Yes	No	Yes	No	* p<0.01			
N (%)	71 (44.4%)	89 (55.6%)	39 (20.1%)	155 (79.9%)	A	B	C	D
Variable	**Median** / *IQR*				Mann-Whitney U Test			
Age (Years)	**49.0** / *19.0*	**41.0** / *20.0*	**54.0** / *17.0*	**44.0** / *18.0*			*	*
Body mass index (kg/m^2)	**30.3** / *4.93*	**26.0** / *3.93*	**31.0** / *6.32*	**24.2** / *5.19*		*	*	*
Waist-to-height ratio	**0.58** / *0.06*	**0.50** / *0.07*	**0.58** / *0.07*	**0.46** / *0.08*		*	*	*
Body fat percentage	**25.8** / *6.00*	**21.6** / *5.65*	**38.3** / *6.50*	**30.5** / *10.0*	*	*	*	*
HbA1c (%)	**5.30** / *0.60*	**5.10** / *0.50*	**5.30** / *0.90*	**4.90** / *0.70*			*	*
Total cholesterol (mmol/l)	**5.50** / *1.70*	**5.20** / *1.30*	**5.70** / *1.00*	**5.30** / *1.40*				
HDL cholesterol (mmol/l)	**1.14** / *0.40*	**1.34** / *0.37*	**1.33** / *0.35*	**1.66** / *0.43*	*	*	*	*
LDL cholesterol (mmol/l)	**3.50** / *1.12*	**3.20** / *1.28*	**3.57** / *0.87*	**3.16** / *1.11*				
Triglycerides (mmol/l)	**1.92** / *1.36*	**1.17** / *0.81*	**1.90** / *0.87*	**0.94** / *0.52*		*	*	*
Variable	**%** / *Frequency*				Fisher Exact Test			
Smoking status								
Never smoker	**25.4** / *18*	**37.1** / *33*	**41.0** / *16*	**35.5** / *55*				
Former smoker	**45.1** / *32*	**28.1** / *25*	**33.3** / *13*	**33.6** / *52*				
Current smoker	**29.6** / *21*	**34.8** / *31*	**25.6** / *10*	**31.0** / *48*				
Obesity						*	*	*
BMI < 25 kg/m2	**4.2** / *3*	**36.0** / *32*	**10.3** / *4*	**59.4** / *92*				
BMI 25 - 29 kg/m2	**45.1** / *32*	**55.1** / *49*	**33.3** / *13*	**29.0** / *45*				
BMI 30 - 34 kg/m2	**38.0** / *27*	**9.0** / *8*	**35.9** / *14*	**11.0** / *17*				
BMI > 35 kg/m2	**12.7** / *9*	**0.0** / *0*	**20.5** / *8*	**0.7** / *1*				
Metabolic syndrome‡								
Abdominal obesity	**90.1** / *64*	**29.2** / *26*	**97.4** / *38*	**38.1** / *59*			*	*
Elevated glucose	**74.7** / *53*	**13.5** / *12*	**48.7** / *19*	**5.8** / *9*			*	*
Low HDL cholesterol	**40.9** / *29*	**6.7** / *6*	**61.5** / *24*	**14.2** / *22*			*	*
Elevated triglycerides	**62.0** / *44*	**21.4** / *19*	**64.1** / *25*	**7.1** / *11*		*	*	*
Hypertension	**97.2** / *69*	**50.6** / *45*	**84.6** / *33*	**19.4** / *30*		*	*	*

† Bonferroni correction for five comparisons. Overall comparison by gender omitted.
‡ According to the harmonized IDF diagostic criteria (Alberti et al., Circulation 2009)

A: MetS (Y) by gender
B: MetS (N) by gender
C: Males by MetS
D: Females by MetS

Table S2: Comparison of tape and 3D body scan measurements

Variable	Manual measurements		Body scan measurements			Delta†		Correlation‡	
	**Median** / *IQR*		**Median** / *IQR*			**Median** / *IQR*		Pearson's *r*	
	M	F		M	F	M	F	M	F
Height	**179.0** / *8.0*	**165.0** / *9.0*		**179.3** / *9.0*	**165.2** / *8.3*	**0.3** / *1.8*	**0.6** / *1.1*	0.990	0.990
Weight	**87.3** / *18.7*	**68.4** / *16.8*		**87.0** / *18.2*	**68.1** / *16.6*	**-0.2** / *0.5*	**-0.2** / *0.5*	0.999	0.999
Right upper arm circ.	**31.0** / *4.0*	**28.0** / *4.6*		**31.7** / *3.2*	**28.7** / *3.8*	**0.9** / *2.2*	**1.0** / *2.0*	0.801	0.896
Waist circumference	**95.5** / *14.7*	**80.1** / *16.9*	*a*	**98.4** / *15.6*	**82.6** / *17.6*	**2.8** / *3.4*	**3.1** / *3.4*	0.968	0.975
			b	**98.9** / *16.6*	**85.9** / *19.5*	**3.5** / *4.0*	**6.1** / *5.1*	0.969	0.962
			c	**101.0** / *14.5*	**92.9** / *14.6*	**6.0** / *4.5*	**12.0** / *5.0*	0.953	0.938
			d	**101.3** / *14.8*	**93.9** / *14.0*	**6.5** / *4.2*	**13.3** / *4.8*	0.956	0.943
Hip circumference	**99.1** / *9.3*	**98.5** / *13.9*	*e*	**100.0** / *13.3*	**98.2** / *15.5*	**2.1** / *6.3*	**1.0** / *4.2*	0.888	0.946
			f	**100.5** / *14.9*	**95.3** / *13.5*	**2.3** / *8.4*	**-3.4** / *5.5*	0.862	0.914
			g	**105.2** / *8.2*	**105.3** / *13.2*	**6.3** / *4.7*	**7.3** / *5.0*	0.886	0.925
			h	**104.1** / *7.9*	**104.3** / *12.8*	**4.9** / *3.5*	**6.0** / *3.6*	0.905	0.937

M: Male subjects (N=160); F: Female subjects (N=194)
All variables measured in centimeter

† Difference body scan measurement - manual measurement; sign test (two-sided) significant for all variables at $p<0.05$
‡ Pearson's correlation coefficient, pairwise by gender, two-sided, with Bonferroni correction; $p<0.05$ for all results

a: High waist circumference; *b:* Waist circumference; *c:* Belly circumference; *d:* Maximum belly circumference
e: Middle hip circumference; *f:* High hip circumference; *g:* Hip circumference; *h:* Buttock circumference

<u>Table S3:</u> Bland-Altman plots

	Male Subjects	Female Subjects
Body Height in cm	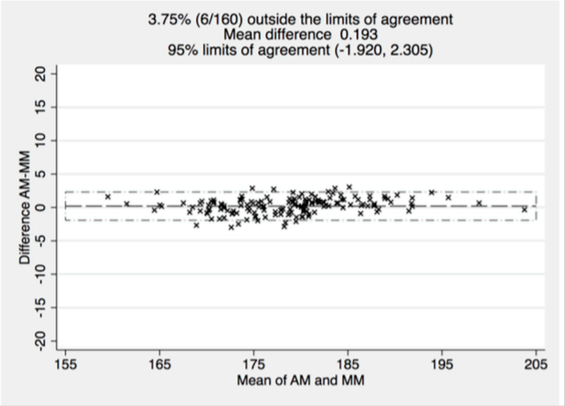	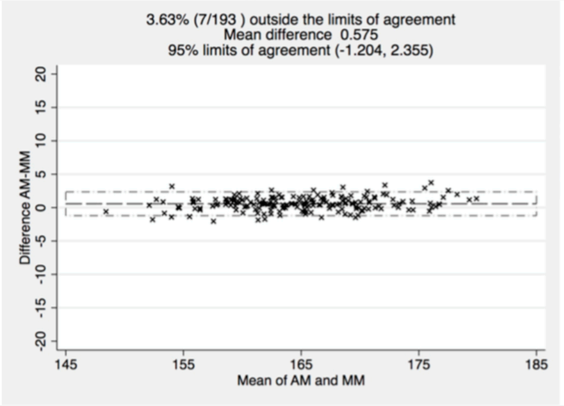
Waist / High Waist Circumference in cm	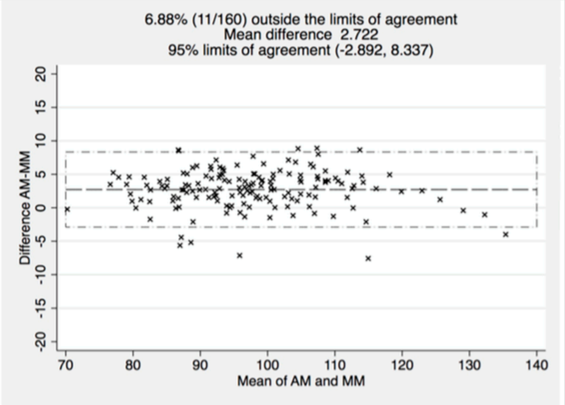	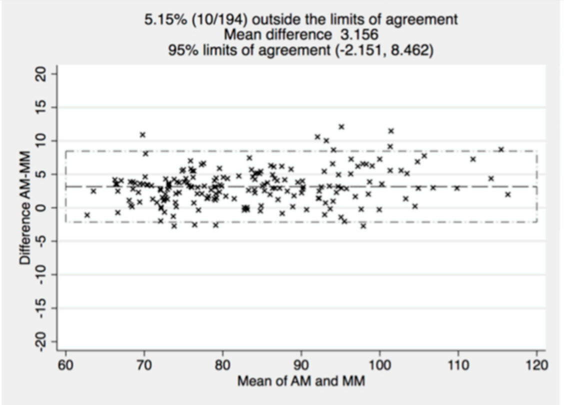
Hip / Middle Hip Circumference in cm	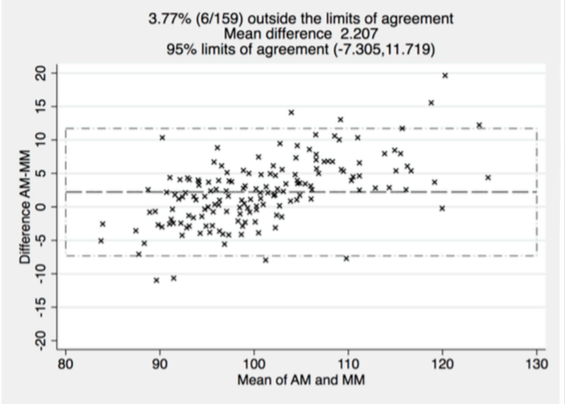	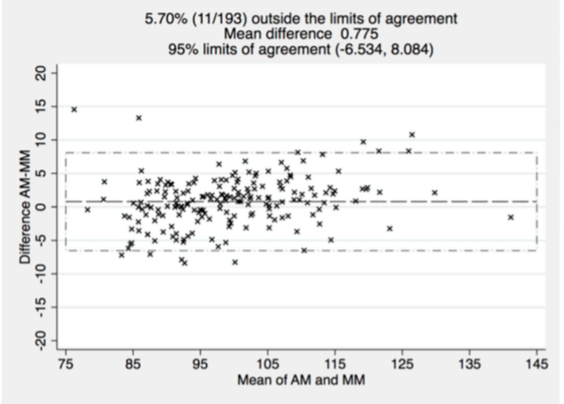
Right Upper Arm Circumference in cm	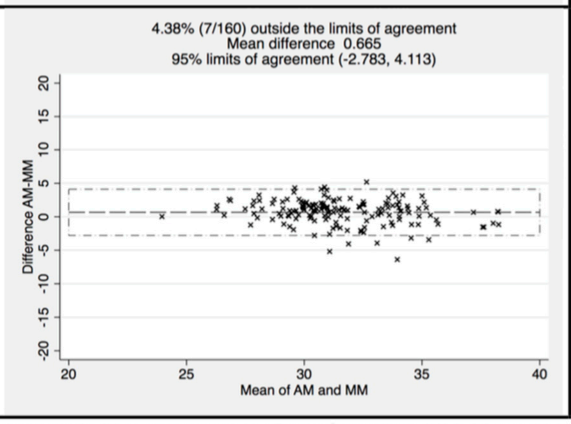	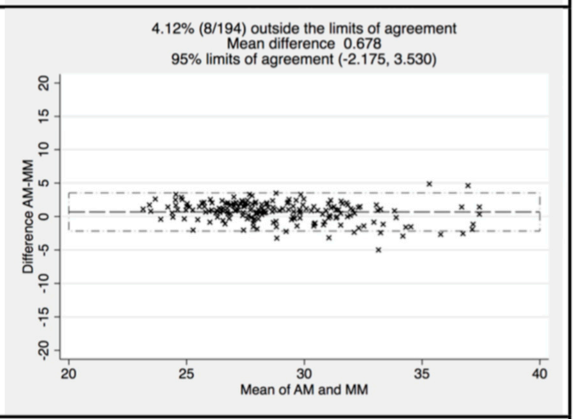

AM: Automatic Measurements by Body Scanning; MM: Manual Measurements;
Dash-Dot-Line: Limits of Agreement; Long Dash: Mean Difference

<u>Table S4:</u> 3D body scan measurements by gender and metabolic syndrome classification

Gender	Male: N=160 (45.2%)		Female: N=194 (54.8%)		Comparisons†			
Metabolic Syndrome	Yes	No	Yes	No	* p<0.01			
N (%)	71 (44.4%)	89 (55.6%)	39 (20.1%)	155 (79.9%)	A	B	C	D
Body scan variable (cm)	**Median** / *IQR*				Mann-Whitney U Test			
Body height	**178.5** / *8.7*	**179.3** / *9.7*	**162.7** / *7.2*	**165.9** / *8.0*	*	*		*
Body weight	**95.8** / *19.4*	**82.6** / *12.6*	**77.8** / *24.2*	**65.2** / *15.2*	*	*	*	*
Middle neck circumference	**43.6** / *4.5*	**40.0** / *3.6*	**38.2** / *4.1*	**33.5** / *3.2*	*	*	*	*
Neck diameter (transversal)	**14.6** / *1.5*	**13.4** / *1.4*	**13.0** / *1.6*	**11.8** / *1.3*	*	*	*	*
Neck circumference at base	**47.9** / *4.9*	**44.6** / *3.7*	**41.6** / *3.8*	**38.7** / *3.3*	*	*	*	*
Sagittal neck diameter at base	**13.3** / *1.8*	**11.5** / *1.7*	**11.5** / *1.5*	**9.8** / *1.5*	*	*	*	*
Bust circumference	**116.1** / *12.9*	**105.3** / *10.4*	**113.1** / *13.1*	**97.5** / *13.2*		*	*	*
Underbust circumference	**109.9** / *10.6*	**98.4** / *8.9*	**97.4** / *13.4*	**82.6** / *11.1*	*	*	*	*
Sagittal chest diameter	**30.5** / *3.7*	**26.8** / *3.2*	**31.7** / *3.8*	**26.5** / *4.3*	*		*	*
Total torso circumference	**184.3** / *8.9*	**176.9** / *9.8*	**166.9** / *13.3*	**161.1** / *8.9*	*	*	*	*
Waist circumference	**108.1** / *13.8*	**93.7** / *11.7*	**101.2** / *15.2*	**82.3** / *16.8*	*	*	*	*
High waist circumference	**107.3** / *13.2*	**92.4** / *11.4*	**97.3** / *15.1*	**79.6** / *14.2*	*	*	*	*
Maximum belly circumference	**109.1** / *16.3*	**96.7** / *9.8*	**105.8** / *14.3*	**91.4** / *12.9*		*	*	*
SAD	**31.4** / *5.2*	**26.0** / *4.3*	**30.0** / *5.2*	**24.1** / *4.6*		*	*	*
High hip circumference	**108.0** / *15.5*	**96.2** / *9.0*	**105.6** / *14.4*	**92.1** / *13.3*		*	*	*
Middle hip circumference	**106.7** / *15.1*	**96.4** / *8.8*	**109.8** / *15.2*	**95.4** / *14.1*			*	*
Hip circumference	**107.8** / *8.4*	**103.3** / *6.8*	**113.3** / *17.1*	**104.1** / *11.4*			*	*
Buttock circumference	**106.6** / *8.7*	**102.0** / *7.3*	**113.0** / *16.1*	**102.2** / *11.8*			*	*
Right upper arm circumference	**32.6** / *3.4*	**31.2** / *2.9*	**31.2** / *4.7*	**28.4** / *3.3*		*	*	*
Right upper arm diameter	**13.8** / *1.9*	**12.7** / *1.7*	**12.4** / *1.9*	**10.6** / *1.7*	*	*	*	*
Right thigh circumference	**58.7** / *5.7*	**56.4** / *4.5*	**58.9** / *9.9*	**56.7** / *5.9*				*

SAD: Sagittal abdominal diameter at maximum belly circumference
† Bonferroni correction for five comparisons. Overall comparison by gender omitted.

A: MetS (Y) by gender
B: MetS (N) by gender
C: Males by MetS
D: Females by MetS

Table S5: Correlation of 3D body scan measurements with metabolic syndrome components

	Metabolic syndrome		MetS comp. without WC†		Abdominal obesity‡		Elevated Glucose		Low HDL cholesterol		Elevated triglycerides		Hypertension		Body fat percentage	
	Spearman's rho§															
Variable	M	F	M	F	M	F	M	F	M	F	M	F	M	F	M	F
Body height																
Body weight	0.48	0.39	0.43	0.35	**0.67**	**0.73**		0.32					0.37	0.30	**0.67**	**0.84**
Transversal neck diameter	0.42	0.39	0.41	0.34	0.52	0.59							0.37	0.31	0.48	0.50
Middle neck circumference	0.57	0.50	0.51	0.50	**0.66**	**0.75**	0.35	0.35					0.45	0.44	**0.60**	**0.74**
Neck circumference at base	0.49	0.38	0.46	0.35	0.58	**0.63**							0.48	0.32	0.53	**0.64**
Sagittal neck diameter at base	0.56	0.47	0.50	0.43	**0.62**	**0.68**	0.37	0.34				0.28	0.45	0.38	0.51	**0.69**
Bust bircumference	0.53	0.53	0.46	0.50	**0.71**	**0.84**		0.38					0.38	0.42	**0.65**	**0.86**
Underbust circumference	0.57	0.52	0.48	0.50	**0.75**	**0.84**		0.37	0.31				0.42	0.46	**0.66**	**0.84**
Sagittal chest diameter	0.53	0.56	0.46	0.53	**0.72**	**0.82**		0.38				0.30	0.41	0.45	**0.66**	**0.85**
Total torso circumference	0.41	0.34	0.40	0.29	0.55	0.57							0.35		0.57	**0.68**
Waist circumference	**0.61**	0.51	0.51	0.49	**0.82**	**0.84**	0.34	0.36				0.29	0.41	0.45	**0.74**	**0.90**
High waist circumference	**0.63**	0.53	0.52	0.52	**0.83**	**0.85**	0.33	0.37				0.29	0.43	0.48	**0.73**	**0.89**
Maximum belly circumference	0.57	0.49	0.48	0.49	**0.79**	**0.83**	0.33	0.36				0.28	0.40	0.44	**0.76**	**0.91**
SAD	**0.62**	0.53	0.53	0.53	**0.80**	**0.83**	0.35	0.39				0.31	0.42	0.47	**0.73**	**0.90**
High hip circumference	0.55	0.49	0.47	0.49	**0.78**	**0.82**	0.33	0.36				0.29	0.38	0.43	**0.76**	**0.91**
Middle hip circumference	0.53	0.47	0.45	0.48	**0.77**	**0.79**	0.32	0.35				0.28	0.37	0.44	**0.75**	**0.91**
Hip circumference	0.35	0.33			0.55	**0.61**		0.29							**0.67**	**0.82**
Buttock circumference	0.38	0.37	0.32	0.33	**0.60**	**0.66**		0.31						0.31	**0.68**	**0.85**
Right upper arm circumference					0.34	0.54		0.29							0.40	**0.66**
Right upper arm diameter	0.32	0.35		0.30	0.39	**0.61**									0.45	**0.64**
Right thigh circumference					0.36	0.48									0.53	**0.68**
Body mass index	0.56	0.49	0.48	0.47	**0.73**	**0.78**		0.38					0.39	0.39	**0.69**	**0.90**
Waist-to-height ratio	**0.62**	0.55	0.50	0.56	**0.74**	**0.83**	0.34	0.39				0.32	0.41	0.50	**0.66**	**0.86**
Waist-to-hip ratio	0.40	0.38	0.38	0.35	0.44	0.56								0.30		0.40
SAD-to-height ratio	**0.63**	0.56	0.52	0.56	**0.74**	**0.80**	0.36	0.41				0.34	0.41	0.49	**0.69**	**0.87**

M: Male subjects (N=160); F: Female subjects (N=194).
SAD: Sagittal abdominal diameter at maximum belly circumference
† Sum of metabolic syndrome components without waist circumference (0-4);
‡ Manually measured waist circumference greater than 94 cm for males and 80 cm for females
§ Spearman's rho, calculated by gender with Bonferroni correction, pairwise analysis
$p<0.05$ for all shown results, statistically insignificant results omitted
